# Treatment of extraarticular knee extension contracture secondary to prolonged external fixation by a modified Judet quadricepsplasty technique

**DOI:** 10.1007/s11751-017-0302-x

**Published:** 2017-12-16

**Authors:** Federico Persico, Oscar Vargas, Gabriel Fletscher, Mauricio Zuluaga

**Affiliations:** Reconstructive Surgery Group, Osteoarticular Diseases Institute, Imbanaco Medical Center, Cali, Colombia

**Keywords:** Quadricepsplasty, External fixation, Extension knee contracture

## Abstract

The goal of this study is to evaluate the functional results of the modified Judet quadricepsplasty for a knee extension contracture secondary to prolonged external fixation. This is a retrospective study of 31 patients with the diagnosis of an extraarticular knee extension contracture who had prolonged external fixation of the femur. Functional assessment was conducted after a minimum follow-up of 1 year. After performing the functional assessment, according to the Judet scale, 51% of the 31 patients had good results and 19.35% (6 cases) showed excellent results. The improvement in mobility from pre-operative to post-operative range of motion was significant. The performance of the technique, following the authors’ described steps and making the subsequent modifications, allowed for partial knee mobility restoration, which significantly improved the patients’ functional status.

*Level of evidence*: IV. Series of cases.

## Introduction

Knee extension contracture is a possible complication after external fixation of the femur, and it can result despite the intensive rehabilitation processes used to preserve the joint mobility. The recommended procedure for the treatment of knee extension contracture is the quadricepsplasty if, following the removal of the external fixator there is persistent limitation of flexion despite intensive physiotherapy [1].

The quadricepsplasty is a surgical technique used to improve flexion for the severely stiff knee. Several procedures are available including the Thompson quadricepsplasty, and the Judet’s technique of quadricepsplasty and its modifications [2]. Due to the technical complexity of the procedure, the need for extensive surgical approaches and the potential complications, these techniques are not popular; in addition, there are only a few reports in the literature [3, 4, 7–9].

The goal of this study is to evaluate the functional results of the modified Judet quadricepsplasty in patients with a knee extension contracture secondary to prolonged external fixation of the femur.

## Materials and methods

This is a retrospective study that reviewed the functional outcomes of patients diagnosed with knee stiffness secondary to prolonged external fixation and treated with Judet’s technique of quadricepsplasty between June 2006 and January 2015. All the patients were older than 18 years, had prior femoral pathology at the time of the intervention and had been treated with an external fixator. The minimum follow-up was 1 year post-operatively. Patients excluded were those with pathological bone fractures, those who did not adhere to the rehabilitation protocol after surgery as described by the technique, or those who had a quadricepsplasty via an arthroscopic release. An ethics committee approval by the institution was obtained prior to reviewing the medical records.

Each patient was assessed  by the authors and asked to provide written consent to analyze and include their clinical information in the study. Descriptive variables included age, sex, indication for external fixation treatment, femoral segment affected and the type of external fixation. The information was stored on an Access database. The external fixation times, length of rehabilitation after the removal of the fixator and the time from the initial injury to the quadricepsplasty were calculated.

The knee range of motion was recorded at the time of clinical assessment and compared to the pre-operative and intra-operative measurements. The final range of motion was used to the determine functional outcome according to the Judet classification:

Excellent: More than 100°;
Good: 80°–99°;
Acceptable: 50°–79°;
Poor: Less than 50°

In addition, the incidence of intra- and post-operative complications was recorded [9, 12].

## Surgical technique

**Fig. 1:**
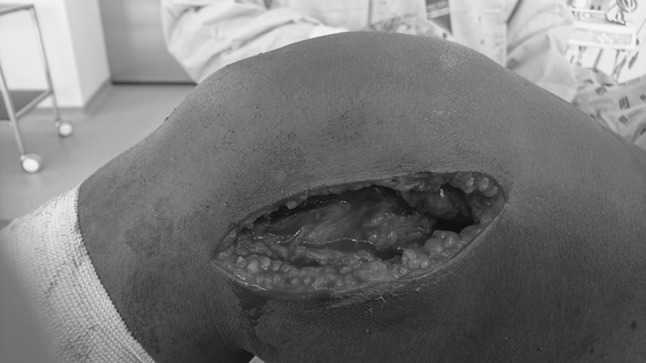
First incision in the medial parapatellar region of the knee

**Fig. 2:**
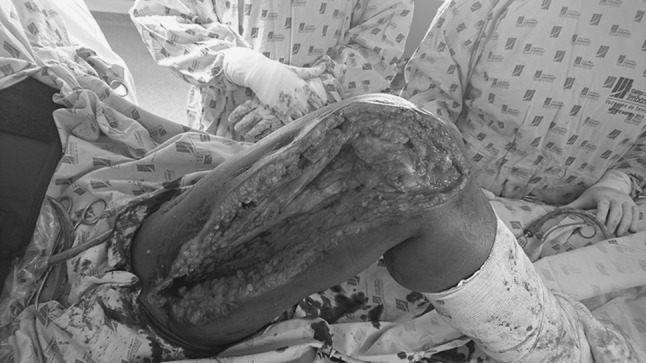
Second incision from 5 cm distally of the greater trochanter to the lateral region of the lower pole of patella

The surgical technique is divided into three phases. In the first phase of the surgical procedure, two incisions are made. The first incision is made from the medial parapatellar aspect of the knee and extended to the medial side of the tibial tuberosity; this allows for access to the patellar tendon by releasing the medial retinaculum, entry into the suprapatellar synovial bursa and freeing of any intra-articular adhesions. During this step, sectioning of the medial collateral ligament is performed (Fig. 1). The second incision is made 5 cm distal to the greater trochanter and extended to the lateral region of the lower pole of patella, through which the lateral retinacular tissues are released thereby ensuring the easy removal of the patellar adhesions to the femoral condyles. The incision allows for release of the vastus lateralis from the linea aspera. The vastus intermedius is freed up also from the front surface of the femur. In the second phase, any redundant bone, e.g., excess bone callus, is removed. The third phase is performed to relax the vastus lateralis proximally in the greater trochanter region and, if necessary, the rectus femoris from the iliac region [2, 5] (Fig. 2).

The surgical incisions are closed, with the knee in the maximum bending position, to ensure the least amount of loss of flexion. The goal is to get at least 90°–100° of flexion [11].

**Fig. 3:**
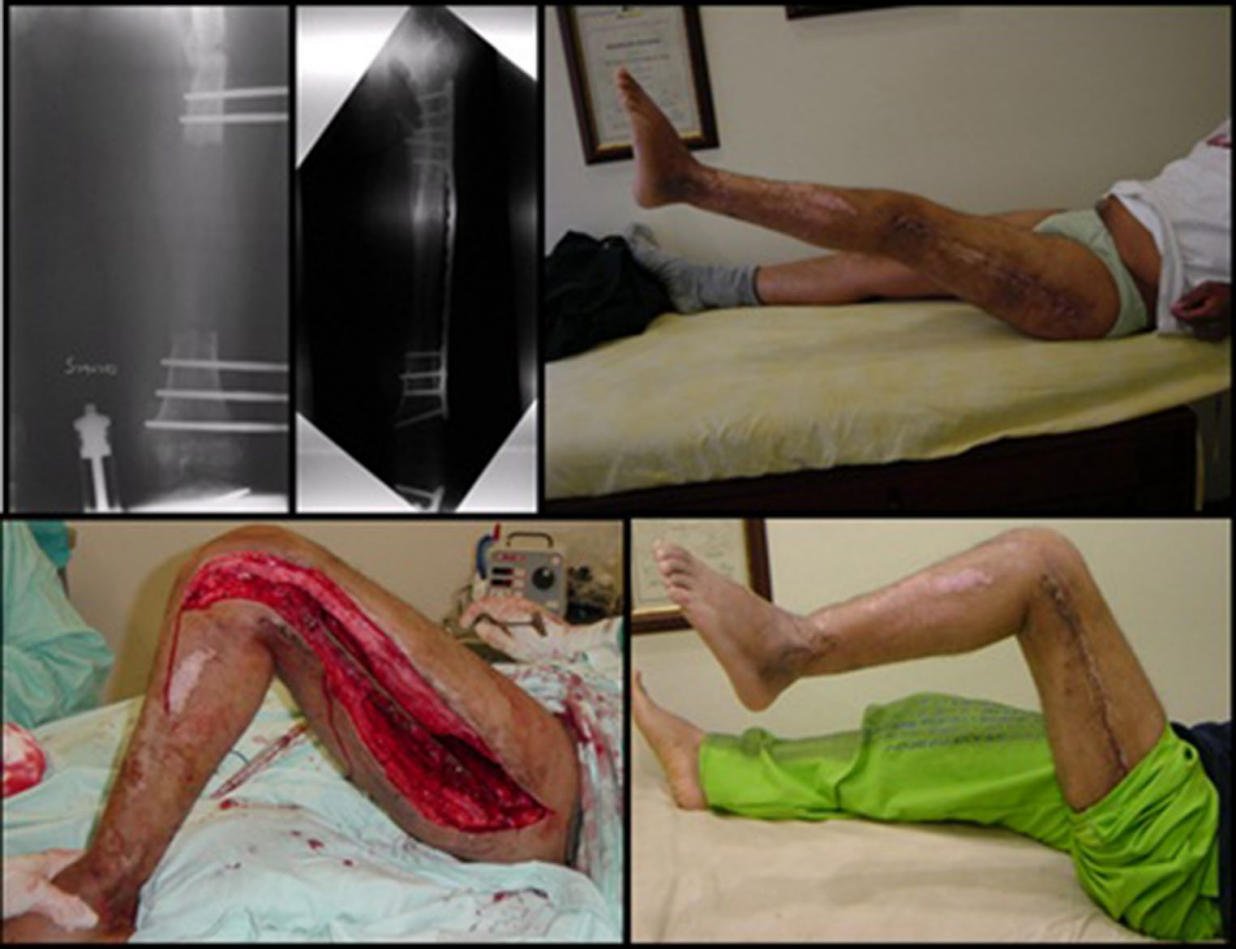
Patient with bone loss secondary to osteomyelitis of the femur. Bone reconstruction with an external fixator. Conversion to plate by lateral approach. Knee extension contracture after bone healing and rehabilitation process. The modified Judet quadricepsplasty was performed. One-year follow-up after surgery showed knee mobility of 0°–90°

In the post-operative period, the hip and knee are bent at 90° for 24 h and continuous passive motion is begun [6]. Active exercises and ambulation are initiated when the patient is comfortable, usually in the first post-operative week. The post-operative period is an important part of the procedure. The patient’s cooperation, adequate control of pain and physical therapy are essential components for obtaining good results from the procedure [1] (Fig. 3).

## Results

The senior author (MZ) performed a modified Judet’s technique of quadricepsplasty on 38 patients over the 9-year period of study; 31 patients fulfilled the inclusion criteria. The group comprised 23 men and 8 women with an average age of 40 years old (range 24–66 years). The main indication for treatment with external fixation was the management of diaphyseal bone infection by excision and bone transport. The average time of external fixation in the study group was 14.1 months, and the post-removal rehabilitation was for 14 months with a minimum of 4 months and a maximum of 60 months (Table 1).

**Table 1:**
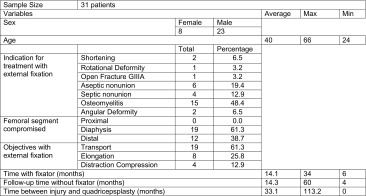
Description of the population

All the patients had a knee extension contracture averaging 17° (0°–50°) pre-operatively. At the time of the procedure, 90.3% of the patients required all three surgical phases. Excellent intra-operative knee motion was achieved in the majority of cases, but this decreased slightly in the post-operative follow-up period. The average follow-up of the patients was 24 months (range 6–30 months). Only two cases persisted with an impaired mobility of the joint. The functional assessment based on the Judet classification showed 51% of the patients had good results and 19.35% (6 cases) were excellent. The improvement in mobility from pre-operative to post-operative range of motion is shown (Table 2).

**Table 2:** Intervention and joint motion

	Total	Percentage
Quadricepsplasty phases		
1	0	0.0
1 and 2	3	9.7
1, 2 and 3	28	90.3
Judet scale		
Excellent	6	19.4
Good	16	51.6
Acceptable	7	22.6
Poor	2	6.5

	Average	Max	Min
Mobility			
Pre-operative	17	50	0
Intra-operative	102	120	100
Post-operative	82	110	45

Patient	Pre-operative	Intra-operative	Post-operative
Knee flexion measurements			
1	20	100	70
2	30	100	80
3	5	110	95
4	15	110	80
5	10	120	110
6	15	100	100
7	10	90	45
8	20	110	80
9	30	115	110
10	5	115	90
11	30	100	90
12	5	90	70
13	30	90	90
14	10	90	70
15	10	100	90
16	15	100	50
18	15	100	110
19	20	80	80
20	25	100	90
21	30	100	60
22	15	110	90
23	50	110	90
24	30	120	100
25	0	100	85
26	30	110	70
27	0	90	80
28	5	100	70
29	10	100	58
30	0	90	60
31	20	100	85

There were no intra-operative complications, but post-operative complications occurred in six patients, being infection the most frequent (Table 3).

**Table 3:** Complications

	Yes	No
Intra-operative	0	31
Post-operative		
Total	6	25
Anaemia	1	
Infection	3	
Haematoma	2	

## Discussion

Several quadricepsplasty techniques have been described. Thompson was the first to describe the quadricepsplasty technique in 1945 followed by Judet with his own technique in 1956. The Judet technique consists of surgical phases for treating knee extension contractures [1]. This technique allows for a gradual release, without disruption of the continuity or integrity of the vastus medialis, vastus lateralis or rectus femoris muscles, and has had more favourable results when compared to the Thompson technique [9]. Since then, various modifications to the initial technique have been described in the literature. Paley modified the Judet quadricepsplasty by emphasizing that parapatellar incisions should extend only to the medial articular line of the proximal tibia, which would allow for the elevation of the medial collateral ligament, minimizing the manipulation of soft tissues and avoiding additional blood loss [10].

The performance of the technique, through a schematic step-focused approach, allows the surgeon to be more attentive to the restrictive elements. In order to achieve a range of acceptable mobility in the evaluated study group, it was necessary to perform all the phases of the technique.

The aim of the intervention is to improve joint motion of the knee, and this is achieved in most of the cases. The Judet classification grades the gain in the flexion after the surgical procedure [9, 12, 13]. Judet documented that his patients had more than 100° of active flexion and only 11% presented a significant decrease in the extension, findings that were substantially better than the results obtained by the Thompson procedure [9]. The findings of our series were consistent with those published in the literature. The loss of extension is not evidenced in our study group.

The complications of the original Judet surgical procedure have been described in up to 23%, including deep sepsis, quadriceps tendon rupture, skin necrosis, fracture of the lateral femoral condyle, wound dehiscence, loss of extension caused by extensive incisions and prolonged surgery or severe post-operative pain [9, 14]. Intra-operative patellar fracture has been documented as a potential complication of the quadricepsplasty technique [8]. In this series, there were no major complications. Infection was the most common complication after surgery and was associated with the decrease in the mobility of the knee. No patient with such complication required additional surgical procedures.

Other techniques have been proposed for improving the mobility of the knee. Blanco et al. [7] developed an endoscopic technique for quadricepsplasty; releasing the muscle adhesions with an aggressive rehabilitation as described by Judet. Hussein et al. described a modification of the Judet technique to perform a limited exposure avoiding transverse incisions in the rectus femoris, and through an incision in the midline of the quadriceps, the rectus femoris was separated from the vastus medialis, lateralis and intermedius in the form of a belt. The vastus intermedius was then separated from the patella, and a manipulation performed after these releases to gain knee flexion [3]. Lee et al. [15] described another modification of the technique, by combining a modified quadricepsplasty and an Ilizarov frame for the management of severe knee rigidity after a diaphyseal fracture around the knee joint which improved the range of motion without any rebound phenomenon.

## Conclusion

The modified Judet quadricepsplasty is a surgical procedure indicated in cases of knee rigidity after prolonged external fixation of the femur. The performance of the technique, following the phases described by the authors and its modifications, allows for restoration of the knee range of motion. Although the potential complications can be challenging, this surgical technique represents an option in the treatment of knee flexion contractures following prolonged external fixation of the femur.
